# A segmented modified look-locker inversion recovery (MOLLI) sequence for high heart rate T1 mapping of mice

**DOI:** 10.1186/1532-429X-17-S1-P120

**Published:** 2015-02-03

**Authors:** Maryam Nezafat, Markus Henningsson, Christian Stehning, Mehmet Akcakaya, Andrea Protti, Rene Botnar

**Affiliations:** Kings college London, London, UK; Philips Research, Hamburg, Germany; Medicine, Beth Israel Deaconess Medical Centre an Harvard Medical School, Boston, MA USA

## Background

Quantitative T_1_ mapping provides myocardial tissue characterization for assessment of various cardiomyopathies. The Modified Look-locker (MOLLI) sequence is widely used for mapping the T_1_quantification, where multiple single-shot images are acquired along the T_1_ recovery curve after an inversion pulse. However, single-shot imaging becomes infeasible for mouse imaging at high heart rates due to motion artifacts and requirements of resolution/coverage. Additionally, typical MOLLI sampling schemes [1] (3-3-5) and the pauses between blocks have to be adapted to the high heart rates in mice. In this work, we propose a segmented acquisition scheme for T_1_ mapping of mouse at high heart rates. After an initial inversion pulse we acquire segments for 20 images in subsequent heartbeats followed by 20 pause heartbeats to allow for full magnetization recovery. The complete k-space is acquired in this fashion over 5 segments per image. Experiments were performed with a T_1_ phantom by simulating high heart rates to evaluate the accuracy of the proposed sequence. Proof of concept T1 maps were also acquired in one healthy mouse.

## Methods

**Figure 1:**
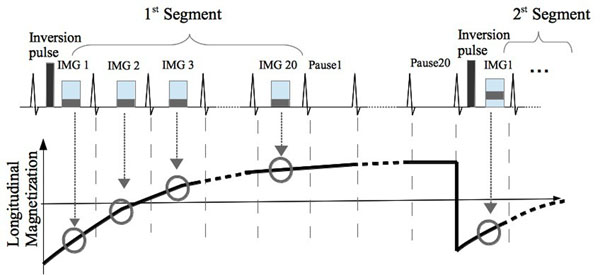
**segmented ECG trigger MOLLI sequence with 20 acquisitions and 20 pauses.** Images were acquired with end-diastole trigger delay. The T_1_ mapping was performed online.

The proposed pulse sequence scheme is illustrated in Figure 1, which consists of a segmented ECG-triggered MOLLI sequence with 20 acquisitions and 20 pauses, which were adapted to the high heart rates. Imaging was performed on a 1.5T Philips Achieva (Philips, Best, The Netherlands) scanner using a 32-element cardiac coil. The phantom consists of 14 vials with T_1_ values ranging from 200 to 1500ms. Data acquisition consisted of a SSFP sequence with the following parameters: TR=2.6 ms, TE=1.3 ms, flip angle=20°, in-plane resolution= 2×2 mm^2^, FOV=210×137 mm^2^. A simulated ECG signal with heart rates of 60, 100, 250, 300 and 400bpm was used. For reference, an inversion recovery spin-echo sequence with 16 different inversion times between 50 and 3000 were used. In vivo mouse imaging was also performed to demonstrate the feasibility of the sequence.

## Results

**Figure 2:**
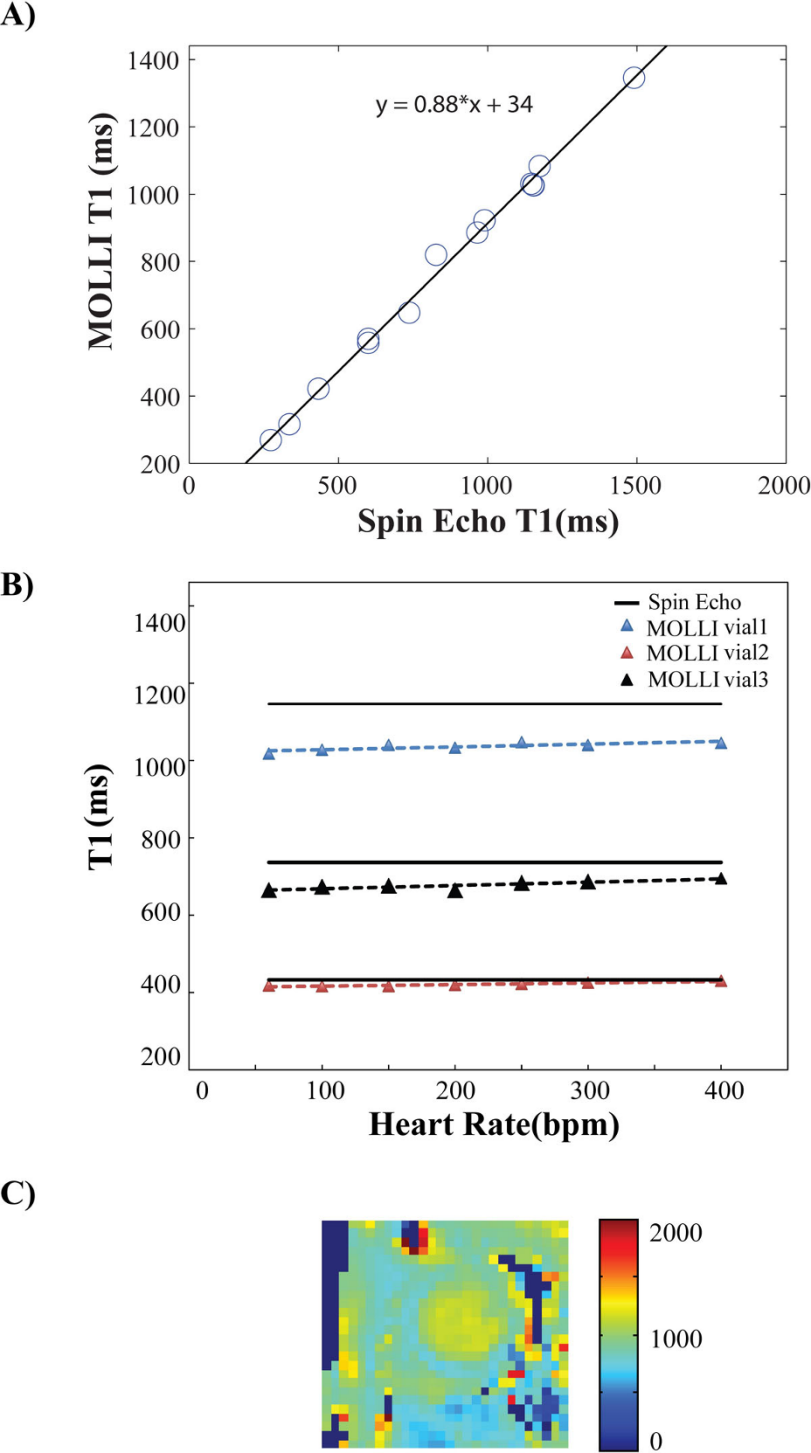
A) Regression analysis of the MOLLI for heart rate of 300bpm and flip angle 20°. B) T1 time of 3 vials of the phantom calculated with SE and MOLLI at various heart rates. The measured T1 shows an underestimation, which is less pronounced at high rates (R^2^ > 0.8). C) T1 map. The measured T1 of the myocardium is 792.9 ± 95.1.

Figure 2A shows the measured T_1_ with the spin echo and the segmented MOLLI technique for a heart rate of 300bpm. The proposed sequence underestimated T_1_ with respect to the spin echo (p=0.3), but the difference was non-significant. For short T_1_ the relative difference between the reference and segmented MOLLI is 0.5-4.1 % and for long T_1_ it is 7.1-10. % for a heart rate of 300bpm. Figure 2B shows results of vials with short, intermediate and long T1 values that were determined with both methods. For higher heart rates the relative difference between the proposed method and reference was relatively small (p=0.3). Figure 2C is a representative in-vivo T1 map image in a mouse acquired with a heart rate of 310bpm.

## Conclusions

We demonstrate the feasibility of T_1_ mapping for high heart rates observed in mice. The proposed segmented MOLLI sequence provides accurate T_1_ estimates for short T_1_ values, while an underestimation is observed for higher T_1_ values, as typical of MOLLI.

## Funding

British Heart Foundation : RG/12/1/29262.
